# Supplementary material for: Glial Fibrillary Acidic Protein: A Biomarker and Drug Target for Alzheimer’s Disease
Source: Pharmaceutics. 2022 Jun 26;14(7):1354. doi: 10.3390/pharmaceutics14071354 (PMC9322874; doi:10.3390/pharmaceutics14071354)
Supplement: Supplementary file 1 [file pharmaceutics-14-01354-s001.zip › pharmaceutics-1758483-supplementary.pdf]

Supplementary Materials

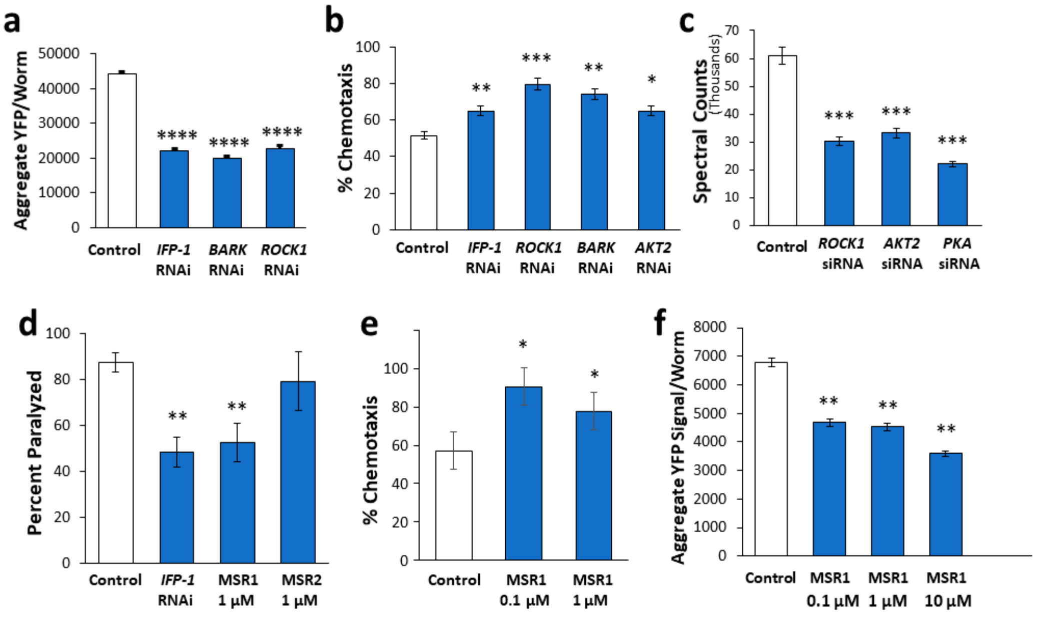

Supplementary Figure S1. MSR1 treatment attenuates aggregates and its associated phenotypic traits in *C. elegans* models of AD. (**a**) RNAi knockdown of *BARK* or *ROCK1* in strain AM141 (a model of Huntington's disease with muscle expression of Q40::YFP), is compared to the effect of RNAi targeting *IFP-1*, the closest C. elegans homolog of *GFAP*. (**b**) RNAi knockdown of *ROCK1, BARK* or *AKT2,* in strain CL2355 (an AD model with pan-neuronal expression of human A$\beta_{1\text{-}42}$). (**c**) RNAi knockdown of *ROCK1, AKT2* or *BARK,* in human T98G cells, reduces the total aggregate burden (measured as total spectral counts for sarkosyl-insoluble material). (**d**) MSR1 protects against aggregation of tau, expressed in muscle of *C. elegans* strain VH255. (**e**) Chemotaxis of *C. elegans* strain CL2355, an AD model with pan-neuronal expression of human A$\beta_{1\text{-}42}$. Chemotaxis to n-butanol was determined for adult worms on day 5 post-hatch. (**f**) Calculated average aggregate fluorescence per worm in *C. elegans* strain AM141 (*q40::yfp*), a model of HD featuring muscle expression of polyglutamine (Q40) fused in-frame to the gene encoding yellow fluorescent protein (YFP). YFP-positive aggregates were counted in adult worms on day 5 post-hatch. (**a – f**) Differences from controls are significant by heteroscedastic, 2-tailed *t* tests at **P*≤0.05; ***P*≤0.005; ****P*≤0.0005; *****P*≤0.00005. Experiments were replicated 3 – 4 times, with consistent results.

Table S1: Kinases predicted to be capable of phosphorylating GFAP sites.

PTM Position	Phosphorylated Amino Acid	Kinase	Peptide Sequence	Actual Score	Cutoff Score
13	S	AGC, ROCK1, or AKT/AKT2	ITSAARR**S**YVSSGEM	27.88	20.90
16	S	ROCK1 or AKT	AARRSYV**S**SGEMMVG	12.02	11.64
323	S	AGC or BARK/GRK	RHVREAA**S**YQEALAR	2.12	2.10
337	S	PKA or PKCα	RLEEEGQ**S**LKDEMAR	119.80	104.65
396	T	BARK/GRK	IRETSLD**T**KSVSEGH	171.89	151.15
398	S	AGC or PKA	ETSLDTK**S**VSEGHLK	21.30	21.27
